# Chi-Squared Automatic Interaction Detection Decision Tree Analysis of Social Determinants for Low Birth Weight in Virginia

**DOI:** 10.3390/ijerph21081060

**Published:** 2024-08-13

**Authors:** Priyadarshini Pattath, Meagan Robinson Maynor, Rexford Anson-Dwamena

**Affiliations:** Virginia Department of Health, Richmond, VA 23218, USA; priya.pattath@vdh.virginia.gov (P.P.);

**Keywords:** chi-squared automatic interaction detection, decision tree analysis, low birth weight, Virginia

## Abstract

This study provides additional context to the literature regarding the social inequities that impact birth outcomes in Virginia using a decision tree analysis. Chi-squared automatic interaction detection data analysis (CHAID) was performed using data from the Virginia birth registry for the years 2015–2019. Birth weight was the outcome variable, while sociodemographic factors and maternity care deserts were the explanatory variables. The prevalence of low birth weight in Virginia was of 8.1%. The CHAID decision tree model demonstrated multilevel interaction among risk factors with three levels, with a total of 34 nodes. All the variables reached significance in the model, with race/ethnicity being the first major predictor variable, each category of race and ethnicity having different significant predictors, followed by prenatal care and maternal education in the next levels. These findings signify modifiable risk factors for low birth weight, in prioritizing efforts such as programs and policies. CHAID decision tree analysis provides an effective approach to detect target populations for further intervention as pathways derived from this decision tree shed light on the different predictors of high-risk population in each of the race/ethnicity demographic categories in Virginia.

## 1. Introduction

The World Health Organization defines low birth weight (LBW) as the birth weight of an infant of 2499 gm (5lb 8.1 oz) or less, regardless of gestational age [1]. LBW is the most important risk factor associated with perinatal and neonatal mortality and morbidity [2]. LBW babies become more susceptible to neurological and respiratory conditions, and future infections and chronic diseases later in life [1,3,4,5]. There are several known individual maternal risk factors and social factors that lead to the outcome of LBW babies. Some of the factors affecting the prevalence of LBW include age, education, income, nutritional status, micronutrients, cigarette smoking, alcohol consumption during pregnancy, substance abuse, and environmental risk factors such as exposure to lead or air pollution [6,7,8]. However, despite the significant literature on the social risk factors for LBW, there are large disparities in LBW outcomes by race, socioeconomic status, and access to prenatal services [9,10]. Disparities in LBW exist among racial groups due to social determinants and socioeconomic status in Virginia [11,12,13]. Improving the health status of pregnant women and infants remains an important public health issue in the Commonwealth of Virginia.

The rate of LBW in Virginia increased from 7.1% in 1990 to 8.4% in 2009 [14]. In 2021, in Virginia, the LBW rate was of 8.3% [12]. Black infants had a higher likelihood to have a LBW than White infants [12]. Several of the social determinants of health risk factors are more pronounced among minority pregnant women [13,15]. Despite several initiatives to prevent risk factors for the delivery of LBW infants in Virginia, health inequities persist [11,12,16,17]. The persistence of the disparities in the infant birth outcomes indicates the need for a deeper understanding of the variables that are highly associated with LBW. Early detection or prediction of outcome of LBW infants in pregnancies may help to prevent LBW.

Several predication tools have been used in the past for determining and identifying the risk factors of LBW. Since birth weight is associated with social factors, and LBW disparities are a substantial public health concern in Virginia, this study seeks to provide additional context to the literature regarding the social inequities that impact birth outcomes in Virginia using a decision tree analysis [18,19].

Data mining tools such as decision tree discovers trends and hidden patterns that help in understanding risk factors and improving quality of patient care, and they have been used for conditions such as breast and ovarian cancer and stroke [20,21]. Few studies have used decision trees for the prediction of infant birth weight [22,23]. The objective of this study is to determine the LBW associated factors in Virginia using decision tree analysis. Based on the Bonferroni test, chi-squared automatic interaction detection (CHAID) is an algorithm analysis of the decision tree model that demonstrates the relationship between variables [18,22]. The CHAID decision tree analysis identifies relationships between split variables and associated related factors, enabling the revelation of population subgroups that are homogenous. CHAID decision tree analysis additionally enables the partition of population into subgroups with different characteristics, and the estimation of prevalence in each subgroup, as opposed to a logistic regression analysis that explores risk factors among the whole population and treats different factors equally. The patterns discovered by the CHAID analysis may improve the quality of the decision-making process for targeted intervention by providing extra ante-natal and prenatal care to the identified sub-population of pregnant women.

## 2. Methods

### 2.1. Data and Variables

This secondary data analysis used data from the Virginia birth registry for the years 2015–2019. The data were geocoded at the census tract level. All live LBW and normal birth weight newborns were included in the study. Socio-demographic characteristics of the mother as reported in the registry, such as age (less than 20 years, 20–24 years, 25–34 years, and above 34 years); race/ethnicity (Non-Hispanic White, Non-Hispanic Black, Non-Hispanic American Indian or Alaskan Native (AIAN), Non-Hispanic Asian or Pacific Islander, and Hispanic); and maternal education (high school and below, some college or associate degree, and bachelor’s degree and above) were used. Based on the literature, being a recipient of special supplemental nutritional program for women, infant, and children (WIC) was included as a socioeconomic variable as a proxy for low income [24]. The WIC special supplemental nutritional program provides nutritional support for low-income pregnant and postpartum mothers and children under the age of five years [24,25]. The WIC recipients were categorized as “Yes”, “No”, and “Unknown”. The literature also suggests that a previous cesarean section may have negative effects on neonatal birth weight and were categorized as “Yes” and “No” for this study [26,27].

Adequate prenatal care was categorized based on the Kotelchuck index (adequate prenatal care, late prenatal care, and no prenatal care) [28]. Access to continuous, high-quality care during the time surrounding pregnancy is the key to prevent birth and maternal morbidity and mortality and achieve positive birth outcomes. Several factors are responsible for inequitable access to maternal healthcare, such as geographic availability and shortage of health workforce, specifically obstetrician–gynecologists (OBGYNs). Family physicians and certified nurse midwifes also affect access during both pregnancy and childbirth due to disproportionate regional shortages [12,29,30,31,32]. Maternity care deserts are counties in which access to maternal healthcare services is limited or absent, either through a lack of services or barrier to access that care [12]. A county is classified as a maternity care desert if there are no hospitals providing obstetrics care, or no birth centers, and no obstetrician or gynecology care providers [12]. 

The Health Resources and Services Administration (HRSA) has designated Health Professional Shortage Areas (HPSAs) related to primary, dental, and mental healthcare. For this study, HPSAs were identified based on Virginia designations [33]. There are 134 counties in Virginia. Disaggregated birth outcome data from the Virginia birth registry for the years 2015–2019 were geocoded at the census tract level to calculate the distance traveled by each mother based on the census tract of residence. A distance matrix of census tract centroid and OB Unit locations was generated. Each mother was linked to the place of residence (census tract) to determine how far she traveled to access services (distance between a place of residence and place of service OB Unit). OBGYN access and drive time in minutes from the population weighted census tract centroid were plotted using geographic information systems mapping software (ESRI, Redland, CA, USA)—Version: GIS ArcPro 3.1. In this study, levels of access to maternity care were set for three levels. OBGYN access within 30 min of drive time from the population weighted census tract centroid was defined as having access to maternity care. OBGYN access over 30 min of drive time from the population weighted census tract centroid was defined as having limited access to maternity care, and OBGYN access over 30 min of drive time from the population weighted census tract centroid, with over 20% population living below 200% FPL and located in a designated HPSA, was defined as maternity care deserts.

### 2.2. Data Analysis

Descriptive, chi-square, and Fischer exact tests were used to compare birth weight and other socioeconomic characteristics. A CHAID decision tree analysis was applied to identify potential factors and determine their relationships with birth weight following univariate analysis. In CHAID analysis, birth weight was the outcome variable, and risk factors were the explanatory variables. The Pearson chi-squared test and maximum likelihood classification were used to compare the categorical variables, which were then classified into binary or more by the predominant significant predictor. Cases in each subgroup were further partitioned by the second most significant predictor in the second step. This analysis continued until the last significant predictors were used [22,23,34]. SPSS version 27 (SPSS Inc., Chicago, IL, USA) was used for the analysis.

## 3. Results

### 3.1. Demographic Profile

A total of 472,878 mothers were included in this study, excluding 46 mothers whose infant’s birth weight was unknown. Table 1 shows the demographic information of the mothers. A majority of the mothers (58.2%) were in the age group of 24–34 years and non-Hispanic White (56.5%). Moreover, 72.5% of the mothers were not WIC recipients, and 38.8% had a bachelor’s degree and above. Most of the mothers reported having adequate prenatal care (95%), access to maternity care (92.6%), and no previous caesarean section (82.7%). A majority of the mothers had infants with normal birth weight (91.9%) (Table 1).

As shown in Table 2, among mothers with LBW infants, 10% of mothers were in the less than 20 years of age category. A remarkably higher proportion of LBW infants were observed in mothers who were non-Hispanic Black (13.2%), and among mothers with a high school or lower education level (9.7%). A higher proportion of LBW infants was reported in mothers with no prenatal care (21.8%), and in mothers who lived in a maternity care desert (9.2%). Of the mothers who had not reported being WIC recipients, 10.5% had LBW infants. 

### 3.2. Chi-Squared Automatic Interaction Detection Decision Tree Analysis of Low Birth Weight

**Figure 1:**
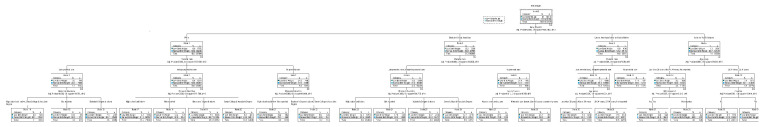
Decision tree analysis on social determinants of low birth weight in Virginia, 2015–2019.

Figure 1 shows the CHAID decision tree analysis for the LBW of infants which are partitioned into statistically significant subgroups. Each node contains node number, “n”, normal birth weight, and low birth weight population in each category. The tree analysis shows the three level CHAID tree with a total of 34 nodes. All the variables reached significance in the model, with race/ethnicity being the first major predictor variable, followed by prenatal care and maternal education in the next levels. In each category, the population of LBW differed significantly (*p* < 0.001, *p* < 0.05). The estimated error of risk in the model was 0.081.

In the years 2015–2019, 8.1% of the population (*n* = 38,514) had LBW infants. The first level of the tree was split into four initial branches based on race/ethnicity. The prevalence of LBW infants among non-Hispanic Black mothers was of 13.2% (Node 2), which was significantly higher than among non-Hispanic White (6.6%, Node 1), Hispanic and AIAN (6.8%, Node 3), and among Asian or Pacific Islander mothers (8.3%, Node 4) (Figure 1).

As seen in the second level of the tree, prenatal care and age group were shown to be the next best predictor variables for each of the LBW splits in the first level. Among non-Hispanic White mothers, those who had no prenatal care had a significantly higher proportion of LBW infants (24.5%) than those who had adequate care (6.4%, Node 6) and late prenatal care (9.2%, Node 5). Among non-Hispanic Black mothers, those who had no prenatal care had a significantly higher proportion of LBW infants (27.9%, Node 9) than those who had adequate and late prenatal care (13.0%, Node 8). Among Hispanic and AIAN mothers, those who had no prenatal care had a higher prevalence of LBW infants (11.4%, Node 11) than mothers with adequate or late prenatal care (6.7%, Node 10) (Figure 1).

Among Asian or Pacific Islander mothers, those in the age group of less than 20 years, above 34 years, and unknown age had a higher prevalence of LBW (9.2%, Node 12) than mothers in the age groups of 20–24 years and 25–34 years (7.9%, Node 13).

Maternal education was the most prominent variable in the third level of the tree for non-Hispanic White mothers. In the subset with late prenatal care, mothers with unknown education had a higher prevalence of LBW (18.0%, Node 15), followed by those with high school and below and some college and associate degree (9.9%, Node 14), compared to mothers with higher education (7.3%, Node 16). This was consistent for non-Hispanic White mothers with adequate prenatal care with unknown education (12.4%, Node 18), high school and below, (8.1%, Node 17), and in mothers with no prenatal care (LBW 29.9%, Node 21).

Among non-Hispanic Black mothers, with late and adequate prenatal care, mothers with unknown education had a higher prevalence of LBW (17.8%, Node 24) compared to those with a high school education level and below, and those with a college degree or a bachelor’s degree and above (12.7%, Node 26, and 10.7%, Node 25, respectively). However, among non-Hispanic Black mothers with no prenatal care, level of access was the predictor in the third level. Mothers living in the maternity desert and those with limited access to maternity care had a significantly higher proportion of LBW (41.7%, Node 28) compared to those with access to maternity care (27.1%, Node 27).

In the category of Hispanic and AIAN mothers, with late and adequate prenatal care, age group was the significant predictor in the third level. Those mothers in the age group of less than 20 years and above 34 years (7.8%, Node 29) had a higher proportion of LBW infants than those in the age group of 20–24 years, 25–34 years, and unknown age (6.3%, Node 30) (Figure 1).

Among Asian or Pacific Islander mothers, in the age groups of less than 20 years, above 34 years, and unknown age, being a WIC recipient was the significant predictor in the third level of the decision tree. These mothers, whose status of being a WIC recipient was unknown, had a higher prevalence of LBW (13.1%, Node 32). Among the Asian or Pacific Islander mothers in the age group of 20–24 years and 25–34 years, previous caesarean section was the significant predictor in the third level. Those mothers who did not have a previous cesarean section had a higher proportion of LBW infants (8.1%, Node 33) (Figure 1).

## 4. Discussion

This study aimed to focus on the most vulnerable population of mothers with LBW infants and social determinant risk factors using CHAID analysis. The results indicate that the prevalence of LBW infants in Virginia was of 8.1% in 2015–2019. The CHAID decision tree model illustrates a multilevel interaction among risk factors through stepwise pathways to detect LBW. The significant variables included race/ethnicity, prenatal care, maternal age, maternal education, level of access, WIC recipients, and previous cesarean section. Risk factors are discussed by hierarchical order as follows.

This study detected that race/ethnicity was the first level of predictor in the decision tree, indicating it as the primary and the most important variable of LBW in Virginia. Non-Hispanic Black mothers had the highest prevalence of LBW infants, which was in accordance with previous studies [9,10,11,12]. Non-Hispanic Black women had poor birth outcomes such as LBW and infant mortality compared to their White counterparts in Virginia [35,36]. These findings highlight the need for healthcare providers to increase education and perceived knowledge on LBW based on race/ethnicity in Virginia [16,17,24,37].

It was interesting to find that each category of race and ethnicity had different significant variables. Prenatal care was significantly associated with LBW for non-Hispanic White, non-Hispanic Black, Hispanic, and AIAN mothers, while age group was significant for Asian or Pacific Islander mothers. Mothers with no prenatal care had the highest prevalence of LBW infants, consistent with previous research [3,5,10]. Inadequate or no prenatal care has been identified as a risk factor for non-Hispanic Black or Hispanic pregnant women for LBW infants [38]. Initiatives in Virginia such as providing targeted prenatal care for at-risk Black pregnant women have been successful in improving birth outcomes such as LBW in the past [16,39]. The findings of the present study are consistent with previous studies and indicate that prenatal care may be a preventive factor of LBW, and it thus appears that pregnant women in Virginia may benefit from a prevention and treatment program and from early enrollment in prenatal care [11].

Interestingly, for Asian or Pacific Islander pregnant women, teenage pregnancies and older women had a higher prevalence of LBW infants. Our findings are consistent with a previous study in Virginia [13]. However, in the earlier study, these results were reported only for non-Hispanic White, non-Hispanic Black, and Hispanic mothers [13]. Younger age or adolescence is a well-established risk factor for adverse birth outcomes such as LBW [40,41]. These findings may assist in prioritizing efforts such as programs and policies that include counseling of young and older pregnant women and unplanned adolescent pregnancies.

A significant finding of this study was the different predictors in the third level of the CHAID decision tree. Education is a known social determinant of health, affecting birth outcomes among pregnant women, with low education contributing to late initiation of prenatal care, consistent with our results [8,42,43,44]. In this study, pregnant women with late or no prenatal care and less education had the highest prevalence of LBW infants for all the race/ethnicities except for Asian or Pacific Islander women. It was also interesting to note that pregnant women with unknown education levels were significantly associated with having LBW infants. This could be explained by misreporting or failure to report. The findings illustrate that social determinants such as education may be an important factor associated with LBW. However, focusing only on one determinant may not achieve the desired result of improving birth outcomes for at-risk mothers. For example, in our findings, less education was significantly associated with LBW for non-Hispanic White pregnant women, while for non-Hispanic Black pregnant women, though low education was significantly associated for women with late and adequate prenatal care, for women with no prenatal care, level of access was significantly associated with LBW.

Access to continuous high-quality maternity care is crucial to improve birth outcomes, where geographic availability of certified midwives and obstetricians plays a significant role [29,31,35,45]. Living in a maternity care desert or region with limited access to care contributes to not receiving prenatal care and may intersect with other social determinants of health, such as poverty level and lack of transportation, leading to poor birth outcomes [32,35,46]. Our finding according to which LBW rate was more prevalent in maternity deserts and areas with limited access is consistent with previous studies for non-Hispanic Black mothers with no prenatal care [32,47,48]. These women may be residing in rural areas, with health workforce shortage and birthing centers [46]. The findings may help develop greater coordination and resource sharing across hospital systems and initiatives that provide logistical and financial support for women needing to travel for care [32].

Age also emerged as a significant factor for Hispanic and AIAN mothers with late and adequate prenatal care. Similar to earlier studies, teenage women and those over 34 years of age had a higher prevalence of LBW infants [13,40,49]. 

Interestingly, for Asian or Pacific Islander mothers, WIC recipient and having a previous cesarean section were significantly associated with LBW in the third level of the CHAID decision tree. In contrast to a previous study, this study showed a higher prevalence of LBW in women without previous cesarean section [26]. However, this significance was only for mothers in the age group of 20–34 years, while other mediating factors such as mother’s clinical history, placenta previa, gestational age, and body mass index were not considered [26].

Income is an important social determinant that impacts pregnancy and its outcomes [8,42,43]. Disparity in maternal nutrition among minority group women and those with low-income level leads to negative birth outcomes [24]. WIC recipients are provided food, nutrition education, and healthcare referrals for low-income pregnant mothers [25]. In our study, younger and older AIAN mothers, and those with unknown reports of being a WIC recipient, were found to have a higher prevalence of LBW infants. This could be explained by the reasoning that these mothers with low income may be unaware of the safety net program of Special Supplemental Nutrition Program for Women, Infants, and Children, or they may not have reported being a recipient. The Virginia Rural Health Plan report states that, in 2014 in Virginia, almost 50% of eligible pregnant women did not participate in the WIC program [50]. This finding highlights the opportunity for providers to refer women for such programs during prenatal care.

This CHAID analysis focusses on hierarchical relations by cross-classification to generate subgroups with different characteristics that deal with significant variables of LBW in Virginia. CHAID decision tree analysis provides an effective approach to detect target population for further intervention. An important finding of this analysis is the different predictors or social determinants based on race and ethnicity. In other words, pregnant women from each race/ethnicity had different social determinants based on different characteristics and interaction among the independent variables. It is highly interesting to note that, among low-income, teens, or above 34 years of age Asian or Pacific Islander women, there is a higher proportion of LBW infants, highlighting the role of age and income. Similarly, among non-Hispanic Black pregnant women with no prenatal care, living in a maternal desert or area with limited access posed a higher risk of LBW infants. It is also noteworthy that, among non-Hispanic Black women, with late or adequate prenatal care, low education was an important factor. The CHAID decision tree analysis detected that, for non-Hispanic White women, low education and having no prenatal care were the most significant risk factors associated with LBW. Having no prenatal care and being a younger or older mother were associated with LBW for Hispanic and AIAN mothers.

The pathways derived from this decision tree shed light on the different factors associated with LBW in each of the race and ethnicity categories in Virginia. These findings have important implications for the state and local health district to provide incentives and subsidies for maternity care health professionals in the maternal desert areas and provide financial support and counselling for women to help in addressing their respective risk factors to reduce the incidence of LBW outcomes. The findings provide public health practitioners, policy-makers, and local organizations with relevant social risk factors for LBW, which can help to inform interventions. The breakdown of the interacting predictors for LBW for different communities makes it possible to highlight the most relevant determinants for a particular group, which may help to reduce the disparity in birth outcomes and for collaborating health and social organizations to address them.

A strength of this study is the use of CHAID analysis as, while multicollinearity may be a concern for regression models, decision trees can handle correlated features effectively due to their inherent nature of recursive splitting and feature selection [18,51]. Studies using CHAID analysis found that the model showed multilevel interactions by showing critical predictors in priority order; therefore, it is possible to detect individual cases showing unique behaviors within the participants that may not be identified using the existing analysis methods [18,51]. Another strength of this CHAID analysis is the large sample size. However, there are limitations to this study. There was no validation of the model; thus, the derived pathways for LBW from this study do not imply causality. The characteristics may have been misreported in the birth registry. This analysis did not account for other factors that may affect LBW outcomes such as maternal health behaviors or medical history. Chronic health conditions such as hypertension, diabetes, heart, lung and kidney problems, and certain medications may lead to having a baby with low birthweight [12]. However, these results suggest considerations to facilitate early prenatal care, counselling, and care for low-income and low-education women among each of the races/ethnicities [9,37,52].

## 5. Conclusions

Despite efforts to reduce the incidence of LBW, it remains a significant public health concern in Virginia. This study identifies several factors for LBW based on the interaction between different predictors. Targeting interventions and channeling resources for prioritizing efforts by focusing on low-income, low-educated, younger, and older Asian or Pacific Islander and Hispanic women, and increasing incentives for healthcare providers in maternal deserts for non-Hispanic Black women, may help in reducing LBW. Not initiating early prenatal care remains a risk factor for LBW, along with low education for non-Hispanic White pregnant women. Future studies that include other maternal medical history-related confounding factors of LBW are suggested to explore the interaction between social determinants and maternal health behaviors.

## Figures and Tables

**Table 1:** Characteristics of population of mothers, Virginia, 2015–2019.

Characteristic	N	Proportion (%)
All	472,878	100
Maternal age *		
<20	14,359	3.0
20–24	79,490	16.8
25–34	275,228	58.2
>34	103,246	21.8
Race/Ethnicity		
Non-Hispanic White	267,046	56.5
Non-Hispanic Black	100,885	21.3
Non-Hispanic, American Indian or Alaskan native	809	0.2
Non-Hispanic Asian or Pacific Islander	35,030	7.4
Hispanic	69,108	14.6
WIC Recipients		
Yes	109,598	23.2
No	343,003	72.5
Unknown	20,277	4.3
Maternal education		
High school and below	171,092	36.2
Some College and Associate Degree	118,226	25.0
Bachelor’s Degree and above	183,560	38.8
Prenatal care		
Adequate prenatal care	449,402	95.0
Late prenatal care	18,329	3.9
No prenatal care	5147	1.1
Level of access		
Access to maternity care	437,725	92.6
Limited access to maternity care	8993	1.9
Maternity care desert	26,160	5.5
Birth weight		
Normal birth weight	434,364	91.9
Low birth weight	38,514	8.1
Previous Cesarean section		
Yes	81,937	17.3
No	390,941	82.7

WIC Recipients—Special Supplemental Nutrition Program for Women, Infants, and Children. * 555 (0.1%) maternal age was missing.

**Table 2:** Univariate analysis of influencing factors of low birth weight, 2015–2019.

Characteristic	Low Birth Weight N (%)	Normal Birth Weight N (%)	*p*
Maternal age			0.000 *
<20	1441 (10.0)	12,918 (90.0)	
20–24	7139 (9.0)	72,351 (91.0)	
25–34	20,898 (7.6)	254,330 (92.4)	
>34	8981 (8.7)	94,265 (91.3)	
Race/Ethnicity			0.000 *
Non-Hispanic White	17,552 (6.6)	249,494 (93.4)	
Non-Hispanic Black	13,318 (13.2)	87,567 (86.8)	
Non-Hispanic, American Indian, or Alaskan native	57 (7.0)	752 (93.0)	
Non-Hispanic Asian or Pacific Islander	2901 (8.3)	32,129 (91.7)	
Hispanic	4686 (6.8)	64,422 (93.2)	
WIC Recipients			0.000 *
Yes	10,481 (9.6)	99,117 (90.4)	
No	25,905 (7.6)	317,098 (92.4)	
Unknown	2128 (10.5)	18,149 (89.5)	
Maternal education			0.000 *
High school and below	16,627 (9.7)	154,465 (90.3)	
Some College and Associate Degree	9692 (8.2)	108,534 (91.8)	
Bachelor’s Degree and above	12,195 (6.6)	171,365 (93.4)	
Prenatal care			0.000 *
Adequate prenatal care	35,636 (7.9)	413,766 (92.1)	
Late prenatal care	1756 (9.6)	16,573 (90.4)	
No prenatal care	1122 (21.8)	4025 (78.2)	
Level of access			0.000 *
Access to maternity care	35,507 (8.1)	402218 (91.9)	
Limited access to maternity care	604 (6.7)	8389 (93.3)	
Maternity care desert	2403 (9.2)	23,757 (90.8)	
Previous Cesarean section			0.044 **
Yes	6530 (8.0)	75,407 (92.0)	
No	31,984 (8.2)	358,957 (91.8)	

* *p* < 0.001, ** *p* < 0.05, WIC Recipients—Special Supplemental Nutrition Program for Women, Infants, and Children.

## Data Availability

Study data are not publicly available. A scientific data request may be submitted to the Virginia department of health.
